# Accuracy of Heart Rate Watches: Implications for Weight Management

**DOI:** 10.1371/journal.pone.0154420

**Published:** 2016-05-27

**Authors:** Matthew P. Wallen, Sjaan R. Gomersall, Shelley E. Keating, Ulrik Wisløff, Jeff S. Coombes

**Affiliations:** 1 Centre for Research on Exercise, Physical Activity and Health (CRExPAH), School of Human Movement and Nutrition Sciences, The University of Queensland, Brisbane, Queensland, Australia; 2 K.G. Jebsen Center of Exercise in Medicine, Department of Circulation and Medical Imaging, Norwegian University of Science and Technology, Trondheim, Norway; University of Las Palmas de Gran Canaria, SPAIN

## Abstract

**Background:**

Wrist-worn monitors claim to provide accurate measures of heart rate and energy expenditure. People wishing to lose weight use these devices to monitor energy balance, however the accuracy of these devices to measure such parameters has not been established.

**Aim:**

To determine the accuracy of four wrist-worn devices (Apple Watch, Fitbit Charge HR, Samsung Gear S and Mio Alpha) to measure heart rate and energy expenditure at rest and during exercise.

**Methods:**

Twenty-two healthy volunteers (50% female; aged 24 ± 5.6 years) completed ~1-hr protocols involving supine and seated rest, walking and running on a treadmill and cycling on an ergometer. Data from the devices collected during the protocol were compared with reference methods: electrocardiography (heart rate) and indirect calorimetry (energy expenditure).

**Results:**

None of the devices performed significantly better overall, however heart rate was consistently more accurate than energy expenditure across all four devices. Correlations between the devices and reference methods were moderate to strong for heart rate (0.67–0.95 [0.35 to 0.98]) and weak to strong for energy expenditure (0.16–0.86 [-0.25 to 0.95]). All devices underestimated both outcomes compared to reference methods. The percentage error for heart rate was small across the devices (range: 1–9%) but greater for energy expenditure (9–43%). Similarly, limits of agreement were considerably narrower for heart rate (ranging from -27.3 to 13.1 bpm) than energy expenditure (ranging from -266.7 to 65.7 kcals) across devices.

**Conclusion:**

These devices accurately measure heart rate. However, estimates of energy expenditure are poor and would have implications for people using these devices for weight loss.

## Introduction

The benefits of participating in regular physical activity are well documented [1], yet physical inactivity remains the largest risk factor for the development of cardiometabolic disease worldwide [2]. Wearable devices have become a popular method of measuring activity-based outcomes and facilitating behavior change to effectuate weight loss [3]. It was estimated that approximately 25 million of these devices would be sold in 2015 and worldwide sales are expected to increase to approximately 12.6 billion U.S. dollars by 2018 [4]. Notably, wrist-worn monitors are predicted to account for 87% of wearable devices shipped in 2018 [5]. These devices claim to provide accurate measures of energy expenditure and, more recently, heart rate via photoplethysmography.

Previous studies investigating the validity of energy expenditure estimates have been limited to devices that do not include a measure of heart rate. These studies have demonstrated moderate validity, typically underestimating total energy expenditure compared to reference methods by approximately 10–30% depending on the device measured [6–9].

With the inclusion of sophisticated photoplethysmography technology, new-generation devices such as the Apple Watch and Fitbit Charge HR have the potential to use heart rate-derived algorithms to contribute to estimates of energy expenditure based on activity intensity [10,11]. Recent evidence suggests this method has acceptable validity, however there is inherent variability, demonstrating that the accuracy of these devices is dependent on the device used, the type and intensity of activity, and skin photosensitivity [12,13]., Melanin concentration and skin pigmentation can attenuate the light wavelength emitted from these devices, thereby reducing pulse rate detection [14]. It is important to recognize, however, that the devices that have previously been evaluated were typically designed for sports performance, and contemporary activity trackers (e.g. Apple Watch, Fitbit Charge HR) have not yet been evaluated.

Given the rapid consumer uptake of these devices, it is critical to determine their accuracy to measure these variables across a variety of modes and intensities given their potential to have a major influence on lifestyle behavior and weight management. The aim of this study was to therefore determine the ability of four popular wrist-worn devices (Apple Watch, Fitbit Charge HR, Samsung Gear S and Mio Alpha) to accurately measure heart rate and energy expenditure during approximately one hour of rest, cycling and treadmill walking.

## Methods

### Participants

Healthy male and female volunteers aged between 18–70 were invited to participate. All participants were recruited from a large metropolitan university. The study received ethical clearance from the University of Queensland (HMS15/2403). Research staff screened all participants for any medical indications that may exclude them from exercise testing and obtained written informed consent. Prior to each visit, participants were advised to refrain from ingesting caffeine and alcohol, and to avoid vigorous physical activity for 24 hours, and from consuming a large meal four hours, prior. A standardised meal replacement beverage (Up & Go, Sanitarium, Australia) was provided to participants two hours before all testing sessions. A 24-hour physical activity and dietary diary was completed prior to the second testing session and participants were asked to replicate these behaviors before the final trial.

### Experimental Protocols

Participants attended the research laboratory on three separate occasions, separated by between 48 hours and 7 days. Visit one included measures of height, weight and skin type assessment via Fitzpatrick Skin Type scale [15]. Maximal oxygen uptake (${\dot{\text{V}}\text{O}}_{2\text{max}}$) via indirect calorimetry (MetaMax 3B, Cortex, Germany) was also assessed using a Bruce treadmill protocol. Standard calibration of gas analysers (two point calibration against room air and known gas concentration of 4.07% CO_2_/15.95% O_2_) and volume (3L Hans Rudolph calibration syringe, Kansas, United States) was performed prior to each assessment as per manufacturers instructions. Measurements of oxygen consumption, carbon dioxide production and minute ventilation were obtained at rest and during exercise.

**Fig 1:**
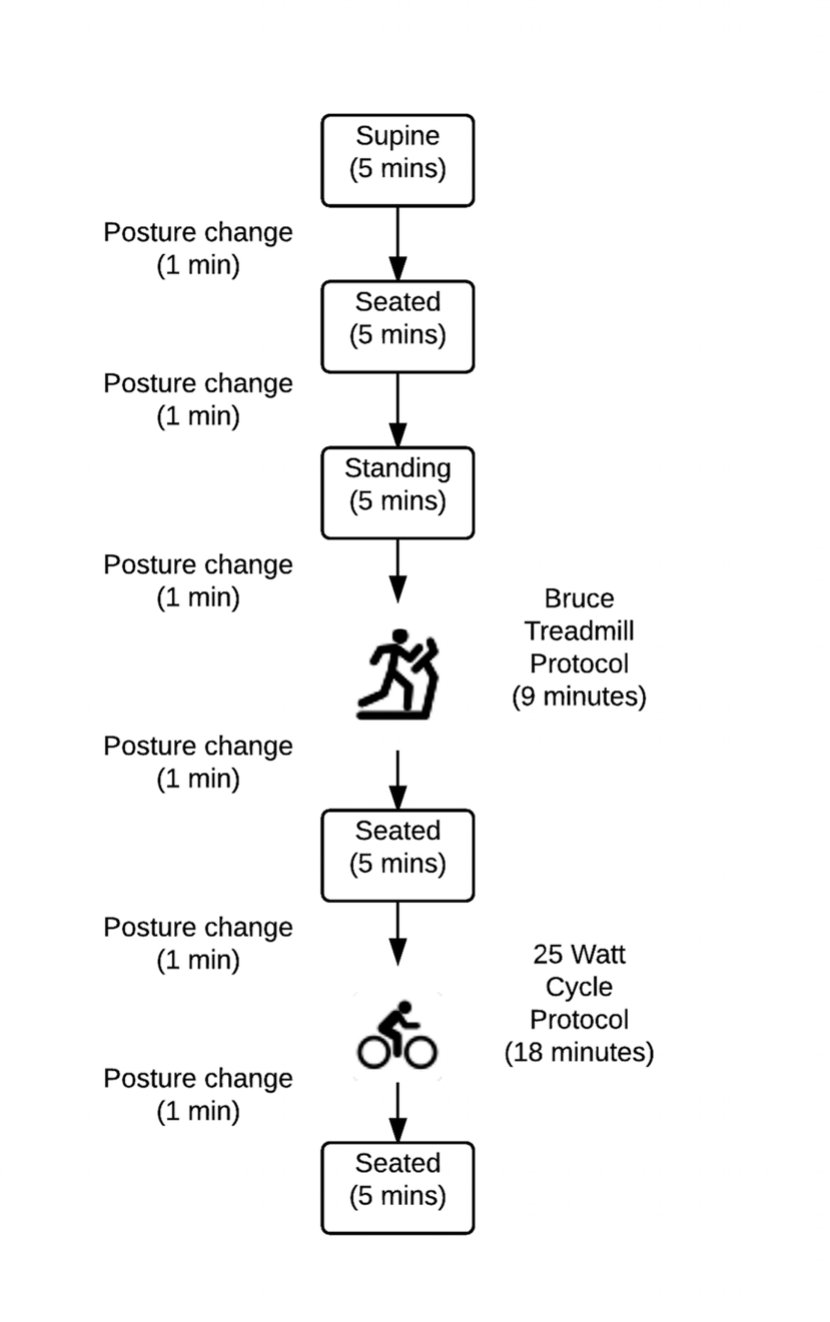
Study protocol (58 mins in total).

Visits two and three were testing sessions, with two devices tested per session (one on each arm), using a randomized and counterbalanced method. Each visit involved the simultaneous recordings of heart rate and energy expenditure from the devices during a range of activities for comparison with reference methods. As three of the devices also measured steps, total steps for the duration of the testing session was also recorded for these devices (Apple Watch, Fitbit Charge HR, Samsung Gear S). To ensure participants were adequately hydrated, urine osmolality was assessed on arrival (Osmocheck Pocket Refractometer, Vitech Scientific Ltd, Tokyo). Activities at rest (lying, sitting, standing) and exercise (walking, cycling) were chosen for the 58-minute protocol (Fig 1). Participants initially performed five-minute periods of supine, sitting and standing, respectively. Three stages of a Bruce graded treadmill exercise protocol were then undertaken followed by five minutes of seated rest. Participants then completed six, three-minute stages of a 25-watt step test (commencing at 25 W) on a cycle ergometer followed by a final five minutes of seated rest.

The devices tested were the Apple Watch (Apple Inc., California, United States), Fitbit Charge HR (Fitbit Inc., San Francisco, United States), Samsung Gear S (Samsung Electronics Co., Ltd., Suwon, South Korea) and Mio Alpha (Mio Global, Canada). As per manufacturer instructions, the devices were individualized for age, gender and anthropometrical data. Devices with compatible smartphone software were synchronized via Bluetooth to an appropriate smartphone to assist with data collection (ease of visualization).

### Reference Methods

Electrocardiography (ECG) electrodes (3-lead, CASE exercise testing system, GE Healthcare, UK) were fitted at each visit and heart rate from the ECG and devices was manually recorded every 15-seconds during the protocol. Energy expenditure was measured using indirect calorimetry with a portable gas-analysis system (MetaMax 3B, Cortex, Germany). Participants were video recorded while walking on the treadmill and step count was determined from the recording retrospectively via visual inspection at half-speed playback.

### Statistical analysis

Pearson (r) or Spearman rank correlation coefficients (rho), for normal- and non-normally distributed data, respectively, intraclass correlation coefficients and Bland-Altman plots with mean bias and upper and lower limits of agreement (LoA) were used to assess criterion validity and agreement between the device and the reference. After visual examination of the plots, systematic bias was assessed using linear regression to determine whether mean difference and/or limits of agreement varied across average values of the device and the reference [16]. Where mean difference and/or limits of agreement varied with average values, estimates were calculated for the mean of the average values. All statistical analyses were conducted using SPSS (Version 22, SPSS Inc.) and data presented as mean ± SD. The strength of correlation coefficients was interpreted based on the following definitions: weak (r = <0.5), moderate (0.5–0.7) and strong (r≥0.7).

## Results

Twenty-two individuals (11 women) volunteered to participate [age: 24.9 ± 5.6 years; height: 173.1 ± 9.9 cm; weight: 72.7 ± 11.8 kg; ${\dot{\text{V}}\text{O}}_{2\text{peak}}$: 50.1 ± 7.8 mL.kg^-1^.min^-1^; maximum heart rate: 189.6 ± 6.9 beats per minute; Fitzpatrick Skin Type scale <IV (n = 15) and >IV (n = 7)]. Participants were euhydrated prior to testing sessions (<700 mOsmol). All participants wore each device once however energy expenditure data were missing for three participants and step count data were missing for two due to a data recording error. Both trials increased heart rate to ~70–80% of maximum with mean oxygen consumption 13.8 ± 1.4 mL.kg^-1^.min^-1^ and 14.3 ± 2.0 mL.kg^-1^.min^-1^ for trial one and two respectively. The mean±SD relative oxygen consumption (mL.kg^-1^.min^-1^) for individual stages of both trials were as follows: supine (5.0 ± 0.7), quiet sitting 1 (4.5 ± 0.8), standing (4.9 ± 1.1), treadmill stage 1 (14.3 ± 1.6), treadmill stage 2 (22.4 ± 2.2), treadmill stage 3 (32.6 ± 3.0), quiet sitting 2 (8.4 ± 2.1), cycling stage 1 (12.2 ± 1.8), cycling stage 2 (14.8 ± 2.3), cycling stage 3 (17.7 ± 2.8), cycling stage 4 (21.5 ± 3.7), cycling stage 5 (25.3 ± 4.5), cycling stage 6 (29.2 ± 5.6), and quiet sitting 3 (7.2 ± 1.3).

Correlations and Bland-Altman findings (mean difference and limits of agreement) are presented in Table 1. No one device performed better overall, however, the outcome of heart rate was consistently more accurate than energy expenditure across all four devices. Correlations between device measures and reference methods varied depending upon the outcome and the device used, ranging from moderate to strong for heart rate (0.67–0.95 [0.35 to 0.98]), and from weak to strong for energy expenditure (0.16–0.86 [-0.25 to 0.95]) (Table 1).

**Table 1:** Sample size, mean, correlation, agreement between device and reference methods and Bland-Altman outcomes for heart rate (bpm) and energy expenditure (kcal).

		Apple Watch	Fitbit Charge HR	Samsung Gear S	Mio ALPHA
**Heart rate**	N	22	22	22	22
**(bpm)**	Device mean ± SD	100.7 ±14.0	92.7 ± 11.5	93.4 ± 13.9	97.7 ± 14.6
	ECG mean ± SD	102.0 ± 14.4	102.0 ± 14.5	100.5 ± 14.6	102.0 ± 13.4
	r/Rho (95% CI)	0.95 (0.88 to 0.98)	0.81 (0.59 to 0.92)	0.67* (0.35 to 0.85)	0.87 (0.71 to 0.94)
	ICC (95% CI)	0.98 (0.94 to 0.99)	0.78 (-0.02 to 0.93)	0.80 (0.40 to 0.93)	0.91 (0.72 to 0.97)
	Mean difference ± SD	-1.3 ± 4.4	-9.3 ± 8.5	-7.1 ± 10.3	-4.3 ± 7.2
	Upper LoA	7.3	7.4	13.1	-0.44.avg + 52.69^†^
	Lower LoA	-9.9	-26.0	-27.3	0.4.avg—61.2^†^
**Energy Expenditure**	N	22	22	19^⌃^	22
**(kcal)**	Device mean ± SD	162.6 ± 33.0	236.8 ± 77.0	261.4 ± 47.5	189.5 ± 95.3
	Indirect calorimetry mean ± SD	285.7 ± 50.2	299.1 ± 46.0	287.5 ± 45.1	290.3 ± 46.3
	r/Rho (95% CI)	0.16 (-0.28 to 0.54)	0.64 (0.30 to 0.84)	0.86 (0.67 to 0.95)	0.46* (0.05 to 0.74)
	ICC (95% CI)	0.05 (-0.05 to 0.17)	0.56 (-0.18 to 0.83)	0.86 (0.15 to 0.96)	0.32 (-0.24 to 0.68)
	Mean difference ± SD	-123.1 ± 55.6	0.61.avg–224.6 ± 59.1^†^	-26.1 ± 24.2	0.91.avg -318.77 ± 84.8^†^
	Upper LoA	-14.1	1.3.avg–334.28^†^	21.3	0.91.avg -318.77 + 166.2^†^
	Lower LoA	-232.1	-0.11.avg–114.92^†^	-73.5	0.91.avg -318.77–166.2^†^

*Notes*: ICC = intraclass correlation coefficient, CI = confidence interval, kcal = kilocalories, ECG = electrocardiography, bpm = beats per minute, SD = standard deviation, avg = average. Correlations r/Rho are Pearson’s correlation coefficient (r) except where indicated by * where they are Spearman rank correlation coefficients (Rho) due to non-normally distributed data.

^†^ Where Bland-Altman parameters were systematically biased (mean difference/limits of agreement), values are presented as linear equations rather than point estimates.

^⌃^ Missing values (n = 3) due to a data recording error.

**Fig 2:**
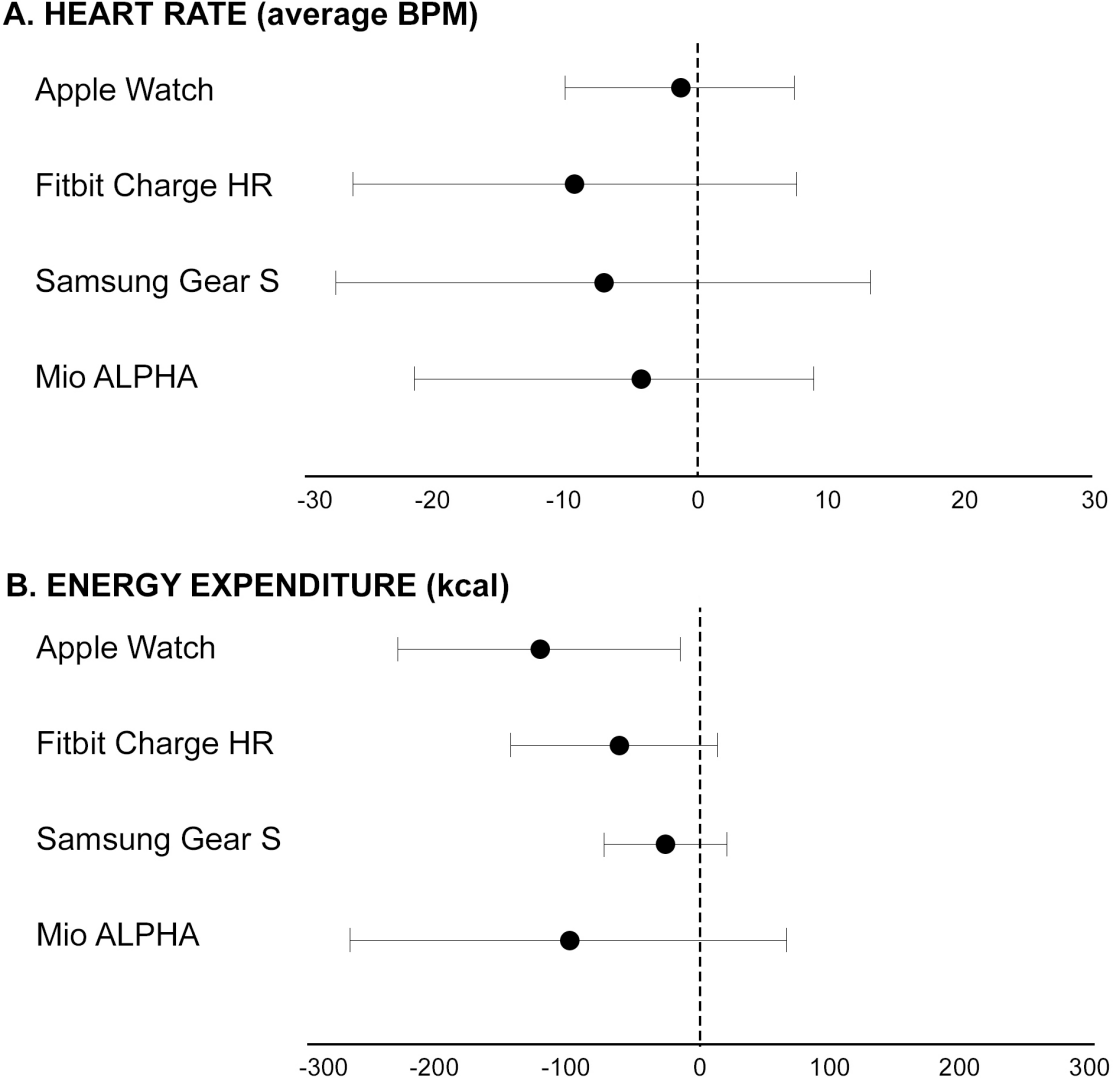
Bland-Altman analyses comparing devices with reference method for (A) heart rate and (B) energy expenditure. Mean difference is indicated by the solid dot, with the lines indicating the 95% limits of agreement. *Notes*: HR = heart rate, kcal = kilocalories, bpm = beat per minute. Where mean difference or limits of agreement were systematically biased, point estimates were calculated using the mean value for the average of the two measures (device and reference).

**Fig 3:**
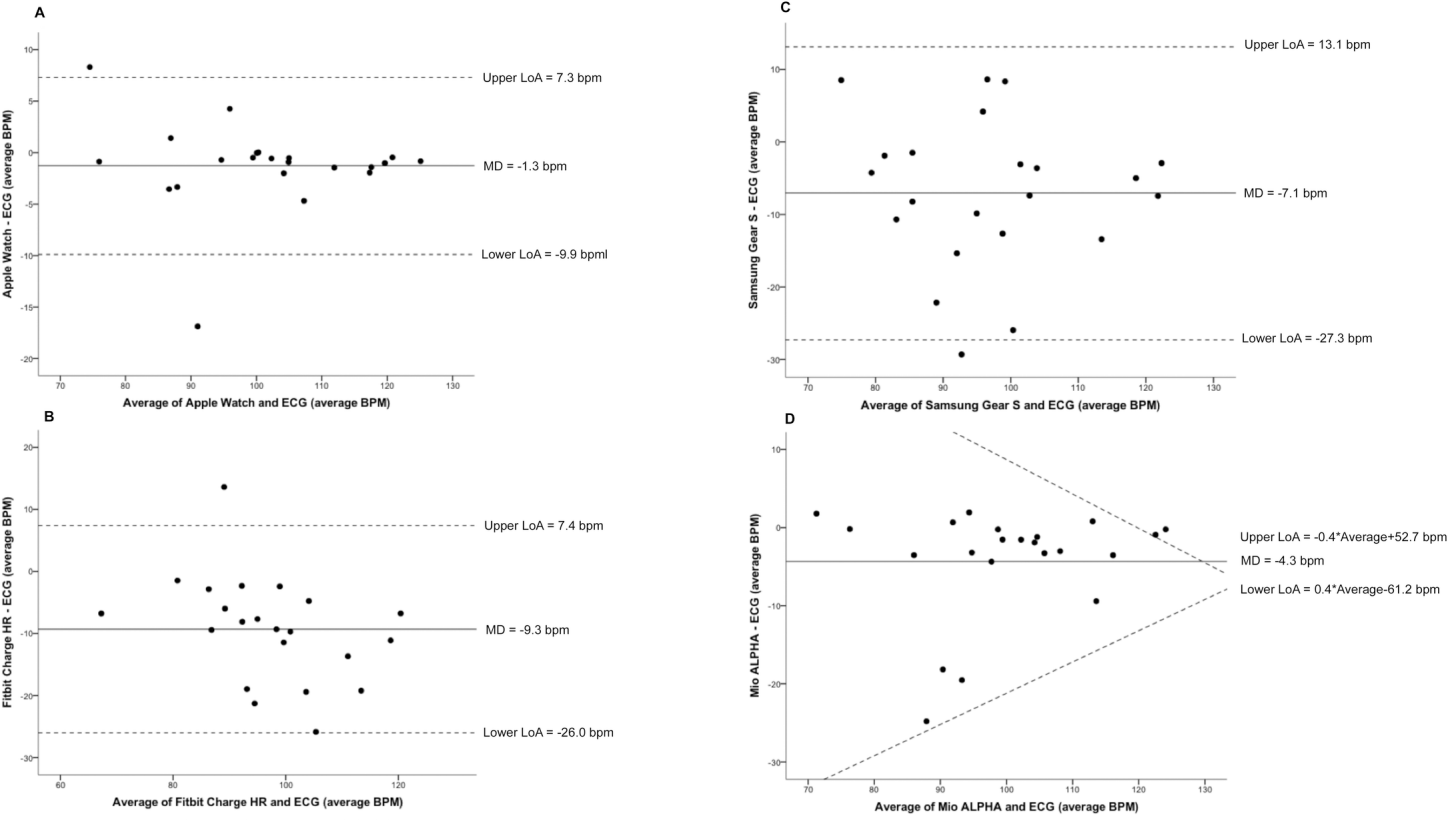
Bland-Altman plots for device [(A) Apple Watch; N = 22, (B) Fitbit Charge HR; N = 22, (C) Samsung Gear S; N = 22, (D) Mio ALPHA; N = 22] and electrocardiography (reference) average heart rate (bpm). The solid line represents the mean difference (bpm) between the two measures and the dashed lines are the 95% limits of agreement (bpm). *Notes*: bpm = beats per minute, LoA = limits of agreement, MD = mean difference.

**Fig 4:**
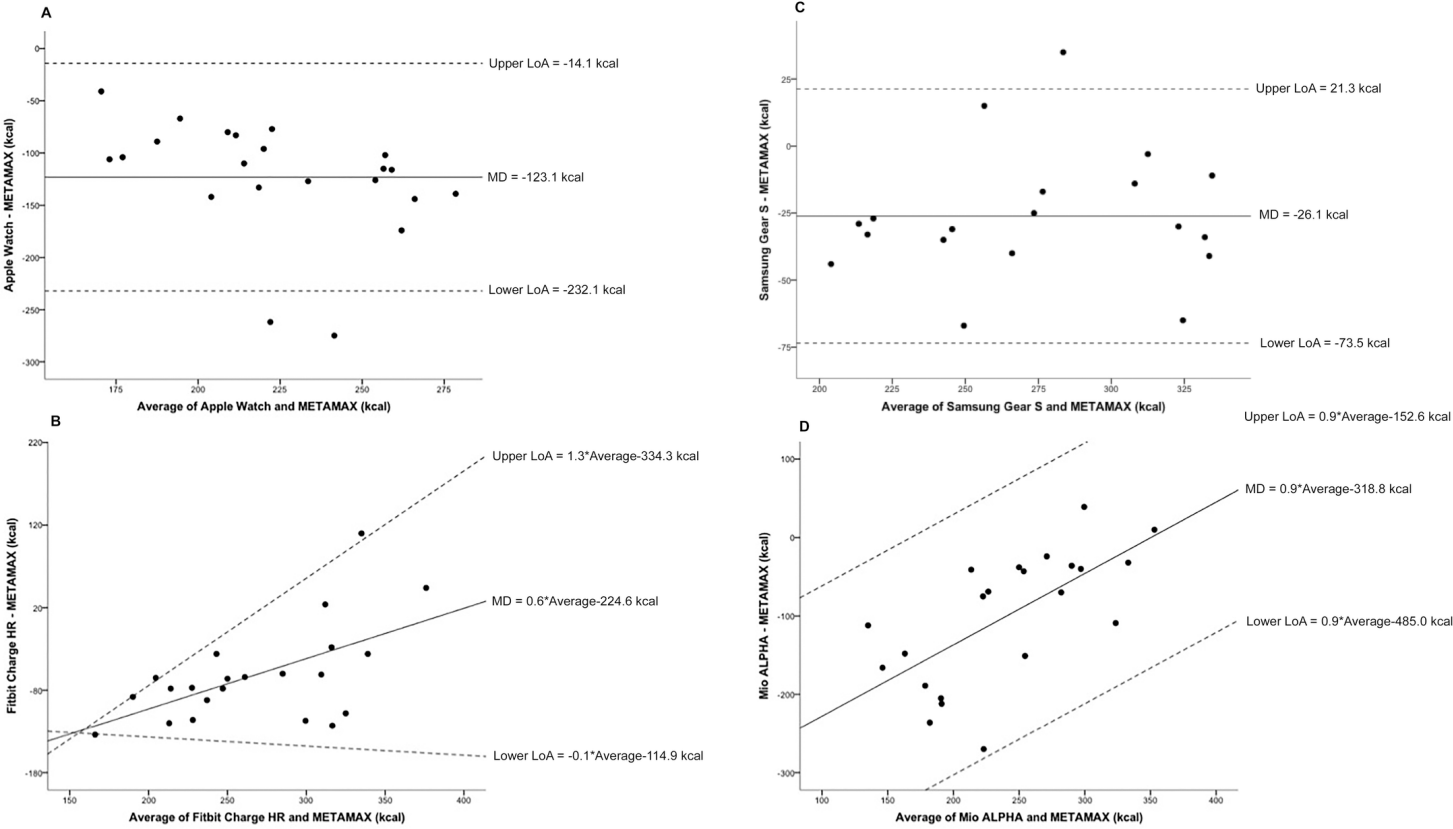
Bland-Altman plots for device [(A) Apple Watch; N = 22, (B) Fitbit Charge HR; N = 22, (C) Samsung Gear S; N = 19, (D) Mio ALPHA; N = 22] and METAMAX (reference) total energy expenditure (kcal). The solid line represents the mean difference (kcal) between the two measures and the dashed lines are the 95% limits of agreement (kcal). *Notes*: kcal = kilocalories, LoA = limits of agreement, MD = mean difference.

Bland-Altman plots indicated that all devices underestimated all outcome measures compared to the reference method (Fig 2). The average underestimation for devices compared to reference methods ranged from 1–9% for heart rate and 9–43% for energy expenditure. The Samsung Gear S demonstrated the greatest variability for heart rate (Lower LoA–Upper LoA; -27.3 to 13.1 bpm) (Fig 3). Furthermore, the Mio ALPHA demonstrated the greatest variability for estimated energy expenditure (-266.7 to 65.7 kcal) (Fig 4). Systematic bias was identified for energy expenditure and heart rate outcomes for the Fitbit Charge HR and Mio ALPHA devices. There were no statistical differences between correlations for heart rate based on skin color (Fitzpatrick Skin Type scale <IV (n = 15) and >IV (n = 7)], except for the Apple Watch, where the correlation for Fitzpatrick Skin Type Scale >IV (r = 1.00) was statistically different to <IV (r = 0.94) (p<0.05).

**Fig 5:**
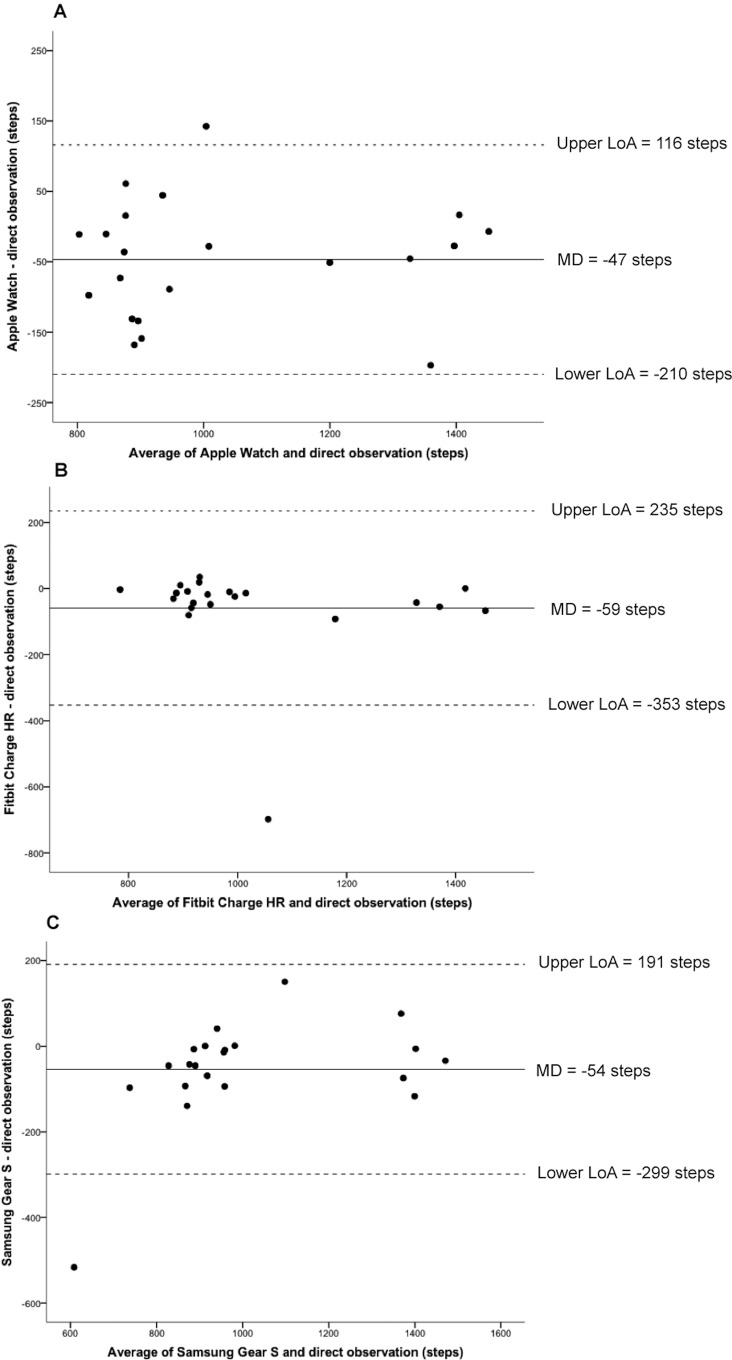
Bland-Altman plots for device [(A) Apple Watch; N = 21, (B) Fitbit Charge HR; N = 21, (C) Samsung Gear S; N = 20] and direct observation (reference) steps. The solid line represents the mean difference (steps) between the two measures and the dashed lines are the 95% limits of agreement (steps). *Notes*: LoA = limits of agreement, MD = mean difference.

Three of the devices measured step count. Correlations between measured steps and the reference method for the Apple Watch (0.70 [0.38 to 0.87]), Fitbit Charge HR (0.67 [0.34 to 0.85] and Samsung Gear S (0.88 [0.72 to 0.95]) were considered moderate to strong. However, the Fitbit Charge HR demonstrated the greatest variability for step count (-353 to 235 steps) (Fig 5). The average error of underestimation for these devices ranged from 4–6%.

## Discussion

This is the first study to examine the accuracy of four popular wrist-worn devices: the Apple Watch, Fitbit Charge HR, Samsung Gear S and Mio Alpha, to measure heart rate and energy expenditure during rest, cycling and treadmill walking. Our findings demonstrate that all devices underestimated heart rate and energy expenditure. No single device demonstrated consistently greater accuracy across these measures and the magnitude of error varied depending on the outcome of interest.

Device estimates of heart rate via photoplethysmography were within 1–9% of reference estimates. Heart rate is commonly used to monitor and prescribe cardiovascular-based exercise intensity [17] and therefore accurate measures are important for precise exercise prescription. Our findings indicate that wrist-worn devices utilizing photoplethysmography offer consumers a convenient and satisfactory method to monitor heart rate while exercising. This is consistent with a recent investigation examining the accuracy of the wrist-based Mio ALPHA, and the forearm-worn Scosche myRhythm, to measure heart rate during rest, exercise and hand-based activities compared to electrocardiography [12]. Overall, the devices had a mean error of <2%, however this varied between the devices for the type of activity. The Mio ALPHA demonstrated the largest mean error during cycling (-4.8%), whilst the largest mean error for the Scosche myRhythm was during walking (-3.13%) [12]. Similarly, Spierer and colleagues (2015) also assessed the accuracy of the Mio ALPHA, and the Omron HR500U during rest, and aerobic and resistance exercise [13]. All devices assessed demonstrated measurement error compared to the reference method, of which was significant during resistance exercise for the Mio ALPHA (mean ± standard error: 23.3 ± 31.94 bpm; p<0.01) [13].

The addition of heart rate measures to traditional accelerometery-based devices that measure physical activity would be expected to improve the accuracy of energy expenditure predictions [10]. However, our findings demonstrate significant variability in the accuracy of energy expenditure estimation, with up to 43% difference between the device and the reference method. As increased energy expenditure through physical activity is recommended as a part of a weight management strategy [18], the inability to accurately estimate energy expenditure is a limitation across these devices. It is difficult to speculate what contributed to errors of this magnitude. It is assumed that each device has a specific algorithm for the determination of energy expenditure. Technical assistance was sought from each company to ascertain information regarding the algorithms used to determine energy expenditure, however this information was not disclosed.

The accuracy of several commercially available activity trackers to measure a variety of physical-activity related outcomes during free-living conditions, which included two wrist-worn devices (Jawbone UP and Misfit Shine) was recently evaluated [7]. Although these devices were not designed to measure photoplethysmography-derived heart rate, the results highlighted that, consistent with our findings, all devices significantly underestimated energy expenditure (Jawbone = -898 kcal; Misfit Shine = -479 kcal), with only a modest association with reference methods (r = 0.74–0.79). Similarly, Sasaki and co-workers (2015) recently validated a hip-worn Fitbit Classic device against indirect calorimetry during a variety of lab-based activities including walking, running and simulated free-living conditions [9]. This study was the first to validate activity-specific estimates of energy expenditure compared to continuous estimates as previous described [6,8]. The Fitbit Classic underestimated energy expenditure for a variety of activities of daily living [-3.1 ± 4.2 kcal/6 min (95% limits of agreement (LoA): -11 to 5.2 kcal/6 min)], locomotion [-5.6 ± 12 kcal/6 min (95% LoA: -29 to 18 kcal/6 min)] and sports [-2.1 ± 12 kcal/6 min (95% LoA -26 to 22 kcal/6 min)]. As increased energy expenditure through physical activity is recommended as a part of a weight management strategy [18], the inability to accurately estimate energy expenditure is a limitation across these devices.

Of interest was the observation that that the Samsung Gear S does not incorporate heart rate into estimations of energy expenditure, whereas the others do. Instead, the Samsung Gear S appears to use an accelerometery-based algorithm during walking/running and predictive equations during cycling. Consistent with previous research [7,19], step count estimates for the Apple Watch, Fitbit Charge HR and Samsung Gear S were acceptable (within 4–6% of the reference).

Limitations of this study included the relatively young and apparently healthy sample of participants (mean: 24.9 ± 5.6 years, range: 19–41 years), and therefore results may not be generalizable to a broader consumer market. Furthermore, the findings associated with laboratory-based protocol cannot be generalized to the free-living context. Finally, it is suggested that the accuracy of these devices may be reduced during higher intensity or resistance-based exercise as a result of movement artefact [13], which was not addressed in this investigation.

## Conclusion

The four devices accurately measure heart rate however estimates of energy expenditure are poor. This limits their use for monitoring energy balance, and therefore as a weight loss aid.

## Supporting Information

S1 Dataset Study data. (XLSX)
